# Metformin Protects Skeletal Muscle from Cardiotoxin Induced Degeneration

**DOI:** 10.1371/journal.pone.0114018

**Published:** 2014-12-02

**Authors:** Francesca Langone, Stefano Cannata, Claudia Fuoco, Daniele Lettieri Barbato, Stefano Testa, Aurelio Pio Nardozza, Maria Rosa Ciriolo, Luisa Castagnoli, Cesare Gargioli, Gianni Cesareni

**Affiliations:** 1 Department of Biology, University of Rome Tor Vergata, Rome, Italy; 2 Fondazione Santa Lucia Istituto di Ricovero e Cura a Carattere Scientifico (IRCCS), Rome, Italy; University of Minnesota Medical School, United States of America

## Abstract

The skeletal muscle tissue has a remarkable capacity to regenerate upon injury. Recent studies have suggested that this regenerative process is improved when AMPK is activated. In the muscle of young and old mice a low calorie diet, which activates AMPK, markedly enhances muscle regeneration. Remarkably, intraperitoneal injection of AICAR, an AMPK agonist, improves the structural integrity of muscles of dystrophin-deficient *mdx* mice. Building on these observations we asked whether metformin, a powerful anti-hyperglycemic drug, which indirectly activates AMPK, affects the response of skeletal muscle to damage. In our conditions, metformin treatment did not significantly influence muscle regeneration. On the other hand we observed that the muscles of metformin treated mice are more resilient to cardiotoxin injury displaying lesser muscle damage. Accordingly myotubes, originated *in vitro* from differentiated C2C12 myoblast cell line, become more resistant to cardiotoxin damage after pre-incubation with metformin. Our results indicate that metformin limits cardiotoxin damage by protecting myotubes from necrosis. Although the details of the molecular mechanisms underlying the protective effect remain to be elucidated, we report a correlation between the ability of metformin to promote resistance to damage and its capacity to counteract the increment of intracellular calcium levels induced by cardiotoxin treatment. Since increased cytoplasmic calcium concentrations characterize additional muscle pathological conditions, including dystrophies, metformin treatment could prove a valuable strategy to ameliorate the conditions of patients affected by dystrophies.

## Introduction

Dietary restriction without malnutrition is proven to extend a healthy average life span by delaying the onset of multiple age-associated diseases in a variety of organisms including primates [1]. Although the underlying mechanisms are not fully understood, the effects are systemic and several organs are targeted by the metabolic perturbation. For instance, in aging muscles, the transcription patterns of metabolic and biosynthetic genes change substantially but most alterations are delayed in mice treated with a low calorie diet [2]. Skeletal muscle plays an important role in maintenance of normal glucose homeostasis, carbohydrate metabolism, locomotion, posture maintenance and breathing. As a consequence, loss of muscle functionality often results in reduced strength, motility and potentially lethal disorders such as muscular dystrophies (MDs) and inflammatory myopathies (IMs) [3].

The link between perturbation of cellular metabolism and muscle function are beginning to be unveiled. Cerletti and colleagues reported evidence that calorie restriction (CR) helps to maintain stem cell function in aging muscles [4]. They observed that mitochondrial abundance and oxygen consumption increased in satellite cells (SCs) from mice on calorie-restricted diet. This metabolic perturbation was associated with an increase in SCs transplant efficiency. Moreover Jahnke and collaborators demonstrated that intraperitoneal injections of AICAR (an AMPK agonist) improve the structural integrity and reduce the degeneration/regeneration of dystrophin-deficient *mdx* mouse muscle. This effect was ascribed to an increase in oxidative metabolism in the AICAR treated muscle fibers [5].

Building on the observation that metabolic reprogramming, which favors oxidative over glycolytic metabolism, has a beneficial effect on skeletal muscle, we asked whether metformin, a powerful calorie restriction-mimicking drug, had also an impact on skeletal muscle damage and regeneration. Biguanides, including metformin and phenformin, have been extensively used for reducing blood glucose levels in type-2 diabetes over the past years [6], [7]. Metformin targets the mitochondrial complex 1 triggering a variety of systemic and cell-specific effects that ultimately lead to a decrease of blood glucose levels [8], which in turns results in AMP accumulation and AMPK activation [9]. Metformin is a pleiotropic drug. Besides its hypoglycemic effect on diabetic patients, metformin treatment has also been associated with a modulation of a variety of additional processes, including neurogenesis [10] protection from cardiovascular [11], [12] diseases and decreased cancer incidence [13]–[15]. In addition Martin-Montalvo and colleagues [16] showed that a long term-treatment with the biguanide enhances the lifespan and health span of mice by delaying aging, increasing antioxidant protection, reducing both oxidative damage accumulation and chronic inflammation.

Although the molecular mechanisms underlying these pleiotropic effects are not well understood, we set out to investigate the effect of metformin treatment on skeletal muscle degeneration and regeneration *in vivo* and *in vitro*. As an experimental system evoking muscle damage we decided to use a well established protocol based on cardiotoxin (CTX) injury [17], [18]. Cardiotoxin is a naturally occurring amphiphilic peptide that interacts with membranes, inhibits protein kinase C (PKC) and induces elevation of cytosolic calcium [19]–[21]. The overload of calcium, in turn, causes cell damages such as cytoskeleton degradation and production of ROS.

Our results show that metformin, rather than promoting SCs activation and fiber regeneration, limits the extent of cytotoxic damage. The protective effect of metformin on muscle damage was confirmed by experiments on C2C12 derived myotubes. We conclude that metformin treatment limits the cytotoxic effect of cardiotoxin by counteracting the increment of intracellular calcium levels induced by the toxin and consequently by reducing necrosis.

## Materials and Methods

### Animal procedures

Nineteen C57BL/6 mice (3 months old) were pre-treated with an intraperitoneal injection of 200 mg/kg body weight of metformin (Sigma Aldrich PHR1084) in PBS or PBS alone (daily for 21 days). For the cardiotoxin muscle-crush injury mice were anesthetized with an intramuscular injection of physiologic saline (10 ml/Kg) containing ketamine (5 mg/ml) and xylazine (1 mg/ml) and then 10 µM of cardiotoxin isolated from *Naja pallida* (Latoxan L81-02) were intramuscularly administered into the tibialis anterior (TA) and gastrocnemius (GC) muscle. PBS and Metformin treated mice were sacrificed 2 days, 5 days and 10 days after cardiotoxin treatment, the TA were collected and snap frozen in OCT for cryosectioning with a Leica cryostat while GC were snap-frozen for protein isolation. Experiments on animals were conducted according to the rules of good animal experimentation I.A.C.U.C. n°432 of March 12 2006 and under ethical approval released on 16/09/2011 from Italian Ministry of Health, protocol #163/2011-B.

### Histological analysis

Evaluation of myofiber area and the fraction of centronucleated myofibers has been carried out by H&E staining. Five 10× randomly selected section of each sample (n = 3) were analyzed with a microscope “camera lucida” in order to draw damaged area. The resulting drawings were analyzed with the ImageJ software for measuring the total area of the section and the fraction of damaged myofiber. Centro-nucleated and embryonic MHC positive fibers were counted considering the damaged area of three sections from each mouse and the results were expressed as the number of centronucleated fibers per 100 µm^2^.

### NADH Transferase Assay

NADH staining has been performed using a standard methodology where the presence of NADH is determined by reduction of nitro tetrazolium blue (NTB) to the water-insoluble purple-blue formazan dye (NTBH2). The tissue section was incubated with freshly made solution B at 37°C for 20 minutes. The stained sample was fixed for 15 minutes in 4% formaldehyde and after extensive wash in ddH_2_O was mounted with Mount Quick Aqueous (BIO-OPTICA). Solution A is Tris/HCl 0,2 M pH 7,4, NBT 4 mg/ml, MgCl_2_ 0,05 M, while Solution B consists of 0,9 ml di solution A; 0,1 ml ddH_2_O; 200 mg/ml NADH.

### Cell culture

The mouse myoblast cell line, C2C12, was purchased from ATCC (American Type Culture Collection, Bethesda, MD, USA) company (CRL-1772). The cells were cultured in growth medium (GM, Dulbecco modified Eagle medium, DMEM), supplemented with 10% fetal bovine serum, (100 U/100 g/ml) penicillin-streptomycin, 1 mM sodium pyruvate and 10 mM HEPES. Cell were differentiated by replacing GM with differentiation medium (DM: DMEM with 2% horse serum) when reached 95% cell confluence. After 5 days, once differentiated, C2C12 myotubes were treated with or without metformin at the final concentration of 0.4, 1 and 5 mM for 24 hours. After 23 hours cardiotoxin was added for 1 hour at the final concentration of 1 µM.

### Immunofluorescence

Cryosections were fixed with 4% of paraformaldehyde (PFA) for 5 minutes, incubated with PBS 1% BSA 0.1% Triton X-100 (1 h at RT), blocked with PBS containing 0.2% Triton X-100 and 20% goat serum (30 min at RT), incubated with the primary antibody (1 h at RT), washed three times and incubated with the secondary antibody (Alexa Fluor conjugated (Life technologies), diluted 1∶200, 1 h at RT). Next, the samples were washed three times and incubated with Hoescht (1 mg/ml, 5 min at RT), washed again and mounted. C2C12 myotubes were fixed with 4% of PFA for 15 minutes and blocked with PBS 1% Serum 10% TritonX-100 0,1% for 1 h at RT. The cells were incubated with the primary antibody 1 h at RT, washed three times and incubated with the anti-mouse secondary antibody Alexa Fluor 488 conjugated (Life technologies A-11001) or anti-mouse secondary antibody Alexa Fluor 555 conjugated (Life technologies A-21425) and anti-rabbit secondary antibody Alexa Fluor 488 conjugated (Life technologies A-11008) diluted 1∶500, 30 min at RT). The samples were washed three times and incubated with Hoescht (1 mg/ml, 5 min at RT). The primary antibodies were: mouse anti-TOM20 (1∶100, Santa Cruz sc-17764), mouse anti-Embryonic MyHC (1∶20, DHSB F1.652), mouse anti MHC (1∶2 MF20, DSHB), rabbit anti laminin (1∶200, SIGMA). Images were acquired with LEICA fluorescent microscope (DMI6000B).

### Immunoblotting

Gastrocnemius treated muscles were homogenized in liquid nitrogen, C2C12 myotubes were washed in plate with ice-cold PBS and both lysed in RIPA lysis buffer (150 mm NaCl, 50 mm Tris-HCl, 1% Nonidet P-40, 0.25% sodium deoxycholate) supplemented with 1 mM pervanadate, 1 mM NaF, protease inhibitor mixture 200× (Sigma), inhibitor phosphatase mixture I and II 100× (Sigma). Samples were incubated on ice for 30 min with the lysis buffer and separated by centrifugation at 14,000 rpm for 30 minutes at 4°C. Protein concentrations were determined by Bradford colorimetric assay (Bio-Rad). Total homogenates were separated by SDS-PAGE. For western blotting analysis, proteins were transferred to membranes, saturated with blocking solution (10% milk and 0.1% Tween-20 in PBS) and incubated with rabbit anti phospho-AMPK (Thr172) antibody (1∶1000, Cell Signaling 2535), rabbit anti-AMPK (1∶1000, Cell Signaling 2603), rabbit anti phospho-RPS6 (Ser240/244) antibody (1∶1000, Cell Signaling 2215), rabbit anti-RPS6 (1∶1000, Cell Signaling 2217), rabbit anti-phospho ACC (Ser79) (1∶1000, Cell Signaling 3661), rabbit anti-ACC (1∶1000, Cell Signaling 3676), rabbit anti-4EBP1 (1∶1000, Cell Signaling 9452), anti-phospho 4EBP1 (Thr37/46) (1∶1000, Cell Signaling 2855) or with mouse anti-Glyceraldehyde-3-Phosphate Dehydrogenase antibody (1∶10 000, Millipore MAB374) overnight at 4°C. The blots were then washed three times and reacted with anti-mouse or anti-rabbit secondary antibody conjugated with HRP (1∶5000, Jackson ImmunoResearch) for 1 hour at room temperature. The blots were then washed three times, and finally visualized with an enhanced chemiluminescent immunoblotting detection system. Densitometric analysis was performed using ImageQuant. Phosphorylated and total proteins were normalized with GAPDH. Then the ratio between phosphorylated and total protein was indicated.

### LDH Activity

After C2C12 myotubes treatment, 50 µl of cell culture medium was incubated with potassium phosphate buffer pH 7,4 containing 18 mM NADH and 72 mM pyruvate. NADH consumption was spectrofotometrically measured at 340 nm. Data were normalized with the total protein contents, expressed as fold change vs control and represent the mean of three experiments ± SE, (*p<0.05, **p<0.01).

### Calcium influx

After 22 hours of metformin pre-treatment, C2C12 derived myotubes were washed twice with Hank's balanced salt solution (HBSS) with 1 mg/ml glucose and 1.8 mM CaCl_2_. C2C12 derived myotubes were incubated with 5 µM Fluo-4AM (F-14201 Life Technologies) for 40 min at RT, washed two times and incubated for 20 minutes in HBSS with 1.8 mM CaCl_2_. Images of calcium influx were obtained using a confocal microscope Olympus FV 1000. Cell cultures were excited with an argon laser at a wavelength of 488 nm, observed with a 10× objective and 2× optical zoom for a total magnification of 20×. Images were scanned at a frame rate of 10 seconds per frame and 50 frames were obtained to record a total of 500 seconds per stimulation. CTX (1 µM) was added during the acquisition, after 50 seconds. Each condition was normalized to the measurements prior to stimulation. Data were expressed as fold change vs control. The plotted results are the average of three independent biological replicas. Peak values for each treatment were combined and analyzed for statistical significance.

Alternatively [22], after treatment with metformin, myotubes were collected, washed twice and suspended in Hank's balanced salt solution (HBSS) with 1 mg/ml glucose and 650 µM CaCl_2_. Cell suspensions were stained with 1 µM Fluo-4AM (F-14201 Life Technologies) for 40 min at RT, washed two times and incubated for 20 minutes in HBSS. Samples were analysed by flow cytometry (FACScalibur; Becton Dickinson), tuned at 488 nm, using the standard band pass filters FL1 (530/30 nm).

### Statistical analysis

All the data presented are mean values ± standard error of at least three experiments. Student's t-test was used to determine significant differences between means in all experiments. The differences were considered significant at P<0.05.

## Results

### Intraperitoneal metformin administration increases the number of TOM20 positive and oxidative fibers

**Figure 1:**
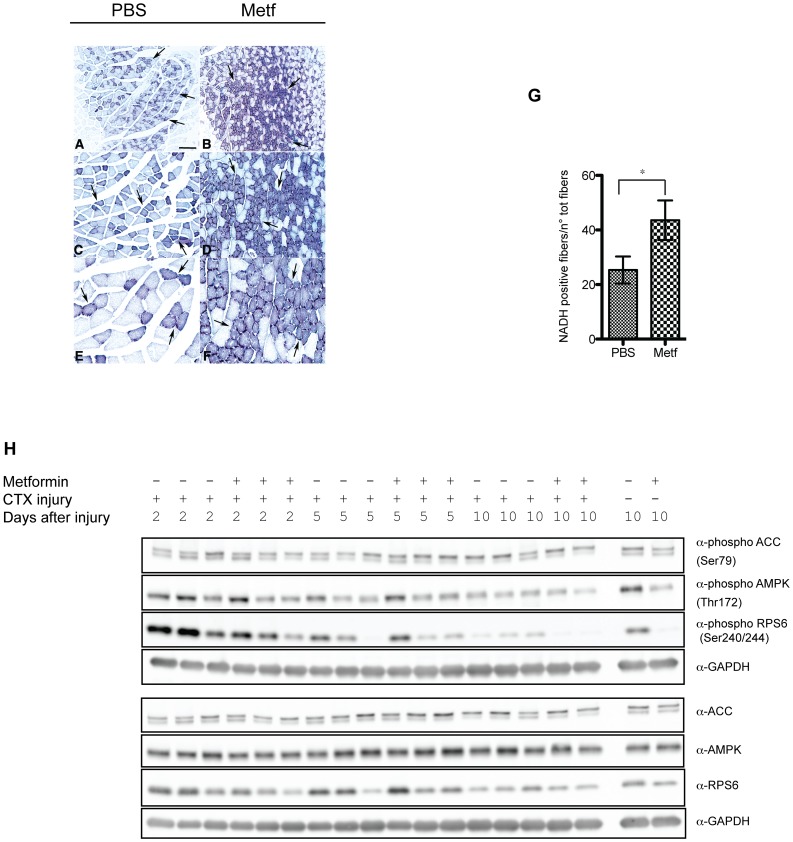
Metformin treatment enhances muscle fiber oxidative metabolism. Histochemical analysis of muscle fibers stained for NADH transferase activity. Panels A to F show three different magnifications of control (A, C, E) and metformin treated fibers (B, D, F). Magnification bar values: (A, B) 500 µm, (C, D) 100 µm, (E, F) 25 µm. (G) The bar graph represents the quantitation of the experiment in A, B, C, D, E, F. Statistical significance was evaluated by the Student's t-test (*p<0.05). (H) Western blot analysis of total AMPK, ACC, RPS6 and their phosphorylation in total protein lysate from metformin-treated or control muscles. GAPDH is used as a loading control.

To study the consequences of metabolic alterations induced by metformin treatment on skeletal muscle damage and regeneration, C57BL/6 mice were treated with a daily intraperitoneal injections of saline solution of metformin (treatment group: n = 9) (200 mg/kg body weight) or of PBS as control (treatment group: n = 9) for 21 days. The extent of damage and regeneration was monitored at 2, 5 and 10 days after intramuscular injection of PBS diluted cardiotoxin (CTX) (10 µM). Suwa and colleagues have reported that metformin treatment, results in an increase of mitochondrial biogenesis in skeletal muscle [23]. We confirmed by NADH transferase staining (Figure 1A–1G) on muscle sections, that metformin treatment significantly increases the number of oxidative (dark) vs glycolytic fibers (pale), when compared to the control, scoring oxidative fibers into five fields selected randomly for each muscle sample (n = 3) (Figure 1A–1G). Remarkably, we observed by immuno-labeling that the mitochondrial specific antigen TOM20 is increased in tibialis anterior (TA) after metformin treatment (Figure S1). TOM20 positive mitochondria-rich green fibers were scored according to the intensity of the TOM20 signal; five randomly selected fields of three different sections from each muscle sample (n = 3) were counted (Figure S1).

ACC inactivation, AMPK activation [9] and the ensuing inactivation of the mTOR pathway [24], are hallmarks of cell response to metformin treatment. Thus, to further characterize the response of muscle tissue to the treatment, we measured the phosphorylation of AMPK, ACC and mTOR and of the downstream target RPS6 (Figure 1H, Figure S1). Quantification of western blots shows that, although the average values are consistent with the expected increment of ACC phosphorylation at Ser79, AMPK phosphorylation in Thr172 and the decrement of RPS6 phosphorylation in Ser240/244, the variations due to metformin administration are not statistically significant.

### Metformin administration protects muscle fibers from cardiotoxin-induced damage

**Figure 2:**
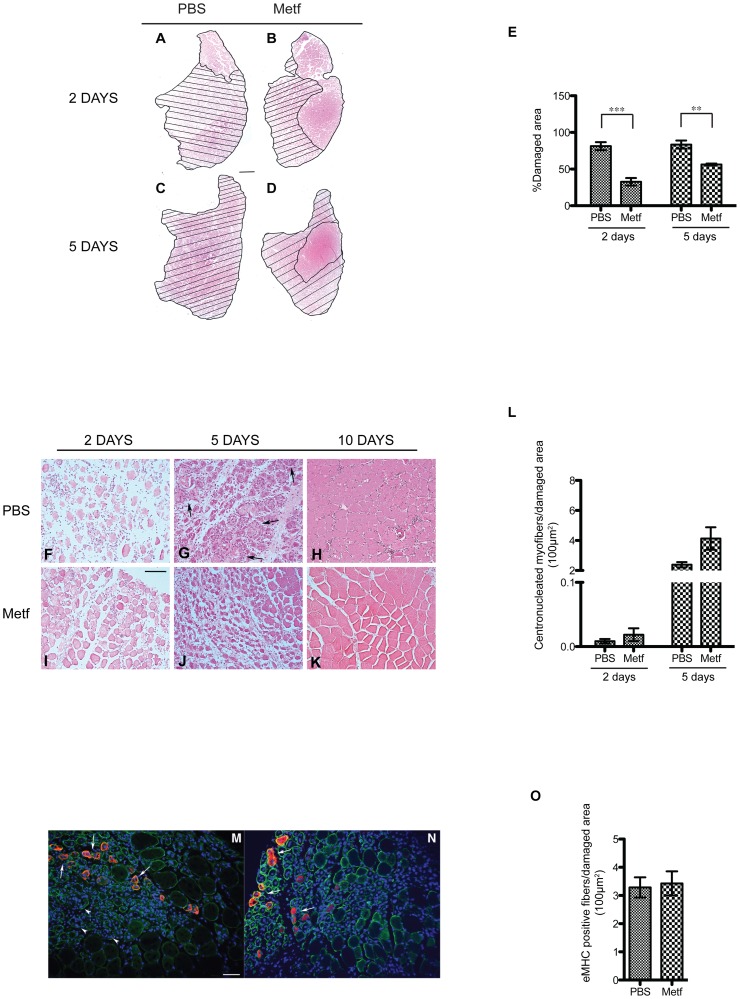
Metformin administration protects muscles from cardiotoxin-induced damage and influences muscle regeneration by reducing cardiotoxin-induced injury. (A–D) Representative images of H&E histological staining on mouse tibialis anterior (TA) section from metformin (B,D) and PBS (A,C) treated mice 2 days (A,B) and 5 days (C,D) after cardiotoxin (CTX) injection. Hatched areas highlight damage caused by intramuscular CTX treatment. (E) Bar graph representing the fraction of injured area. Average values were obtained from five randomly selected sections (magnification 10×) of each sample (n = 3). (F–K) H&E staining on tibialis anterior sections from metformin (I–K) and control PBS (F–H) treated mice at 2 days (F, I), 5 days (G,J) and 10 days (H,K) after CTX damage induction. Arrows in G point to areas of inflammatory cell infiltration. (L) Bar graphs representing the average number of centronucleated fibers per 100 µm^2^ of damaged area 2 and 5 days after CTX treatment, in metformin treated and untreated muscles. Five 20× randomly selected fields of 2 different sections from each sample (n = 3) have been analysed. Values are presented as means ± standard error and statistical significance has been estimated using the Student's t-test (*p<0.05, **p<0.01, ***p<0.001). Scale bars value: (A–D) 500 µm; (F–K) 100 µm. (M,N) Immunofluorescence analysis for Embryonic MHC (red) and Laminin (green) on 5 days CTX damaged TA sections from PBS (M) and Metformin treated (N) revealing regenerating fibers (arrows) and still degenerating myofibers (arrowheads). Scale bar 50 µm. (O) Bar graphs representing the average number of Embryonic MHC fibers per 100 µm^2^ of damaged area 5 days after CTX treatment, in metformin treated and untreated muscles.

To evaluate whether metformin treatment affects the severity of CTX damage, we analyzed hematoxylin and eosin (H&E) stained TA sections of CTX treated mice and we compared the extent of the injured area in the PBS and metformin conditioned mice at two (Figure 2A–2B) and five days after injury (Figure 2C–2D). We observe that metformin pre-treatment significantly reduces the fraction of damaged area at two and five days after CTX treatment (Figure 2E). Moreover H&E staining of TA sections (Figure 2F–2K) showed that, 2 days after CTX injury, metformin-treated mice (Figure 2I) had a significantly lower amount of fibers in degeneration when compared to control mice (Figure 2F). The differences between metformin treated and control mice were more evident 5 days after CTX injection (Figure 2G,2J). In fact, while muscle sections from control mice still showed a considerable presence of inflammatory cells cramming into degenerating muscle fibers and few centronucleated regenerating fibers (Figure 2G), we observed little degeneration in sections of metformin-treated mice muscles. In metformin treated muscles, degenerating fibers were replaced by regenerating tissue, characterized by smaller fiber size and by the presence of centronucleated fibers (Figure 2J). Ten days after damage, little evidence of damaged tissue was still present and normal muscle morphology was restored both in metformin treated and untreated muscles (Figure 2K,2H). However, a few cell infiltrates were still observed in untreated control mice (Figure 2H). Low calorie diet has been reported to increase regeneration rate [4]. Thus we checked whether, besides the observed protective effect on muscle degeneration, metformin had also any effect on regeneration efficiency. Regenerating fibers can be identified by the presence of nuclei that are located in the center of the fiber and not at the periphery, as in mature fibers. To this end we measured the number of centronucleated fibers in metformin treated and untreated mice (Figure 2L). Five days after CTX-induced damage the average number of centronucleated myofibers per area of damaged tissue is marginally larger in metformin conditioned mice than in controls (p value = 0.067). To further describe muscle regeneration, we stained muscle sections with anti embryonic MyHC (eMHC) antibody and we counted the positive fibers (Figure 2M–2O). Five days after CTX-induced damage the average number of eMHC positive fibers per area of damaged tissue in metformin treated mice is not significantly higher than in control mice.

### Metformin treatment attenuates cardiotoxin damage in *in vitro* generated myotubes

**Figure 3:**
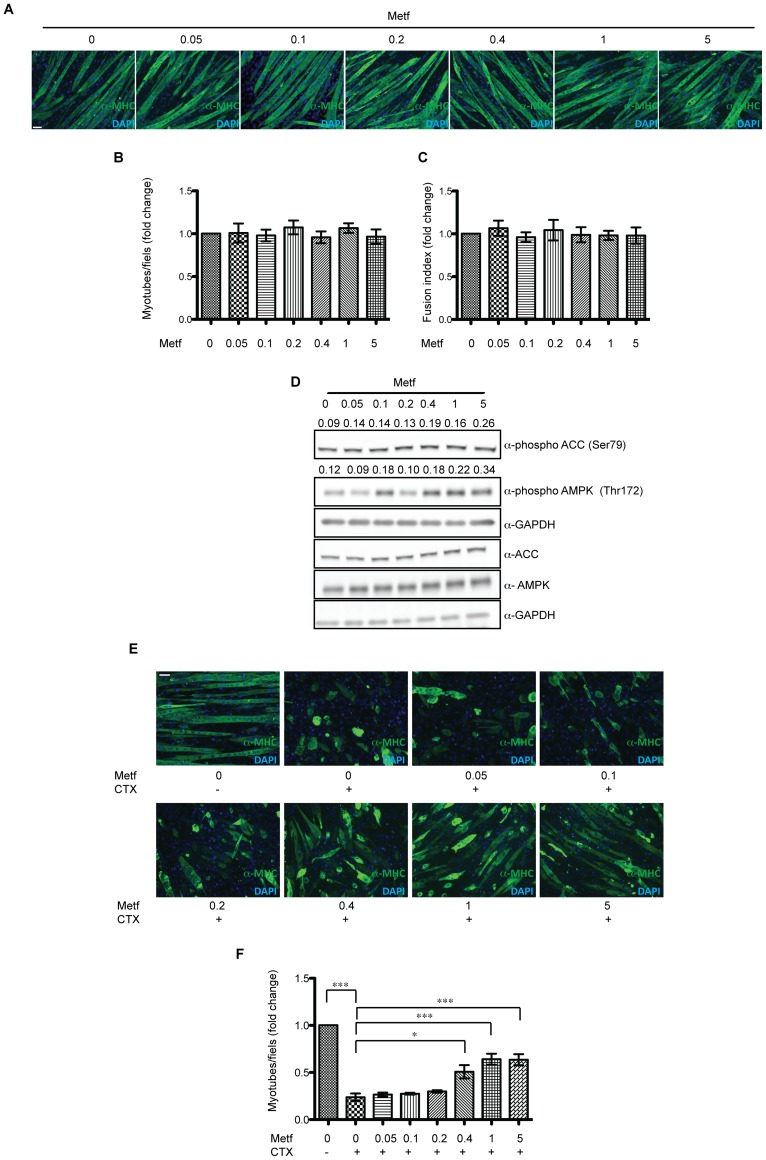
Metformin treatment attenuates cardiotoxin damage of C2C12 myotubes. (A) C2C12 cells were plated at 95% cell confluence and induced to differentiate by replacing growth medium with differentiation medium. After appearance of myotubes the samples were treted for 24 hours with metformin (0.05, 0.1, 0.2, 0.4, 1, 5 mM) and stained with an antibody for myosin heavy chain (MHC). (B) The bar graph illustrates the average number of C2C12 myotubes/field after metformin treatment at the indicated concentrations. The values are the mean of three independent experiments ± SE. (C) Average fusion index (ratio between the number of nuclei inside the myotubes and the total number of myotubes), after metformin treatment with different concentrations of metformin (0.05, 0.1, 0.2, 0.4, 1, 5 mM) for 24 h. (D) Myotube extracts before and after treatment with metformin were electrophoresed on acrylamide gel and after blotting, the phosphorylations of AMPK in Thr172, of ACC in Ser79, of RPS6 in Ser240/244 were revealed with specific antibodies. GAPDH serves as loading and normalizing control. Numbers above each band represent the densitometric analysis. (E) C2C12 derived myotubes were treated as described above, after 1 hour exposure to cardiotoxin, the myotubes were observed under a fluorescence microscope. The samples were labeled with an anti-MHC antibody and a secondary Alexa Fluor 488 conjugated antibody. (F) The graph illustrates the average number of C2C12 myotubes after metformin treatment. Data are the mean of three experiments ± SE (*p<0.05, **p<0.01, ***p<0.001). Scale bars value: (A,E) 50 µm.

In order to confirm the activity of metformin on limiting CTX-induced muscle damage in the absence of “confounding” factors (inflammation, regeneration), we tested the effect of metformin on myotubes generated *in vitro* by differentiating the myoblast cell line C2C12. *In vitro* generated myotubes were treated with metformin at different concentrations (0, 0.05, 0.1, 0.2, 0.4, 1 and 5 mM) for 24 hours. We first monitored whether metformin affects the number of C2C12 derived myotubes and their fusion index (Figure 3A–3C). No significant effect was observed after treatment with metformin. Western blot analysis (Figure 3D) revealed that, metformin concentrations of 0.4 mM or higher are able to induce the phosphorylation of AMPK and ACC. In order to verify if the high metformin concentrations could inhibit protein synthesis via mTOR pathway and give rise to confounding factors we checked the mTOR pathway and in particular we studied the phosphorylation of downstream proteins RPS6 [24] and of the translation initiation inhibitor 4EBP1 [25] (Figure S2). We did not observe any dramatic difference in the phosphorylation of these proteins and therefore we conclude that metformin does not significantly affect myotube protein synthesis via the mTOR RPS6 pathway.

To verify metformin protection of C2C12 myotubes damage by CTX, 23 hours after metformin exposure we treated myotubes with 1 µM CTX for 1 hour. The decrease in the number of undamaged myotubes, after cardiotoxin treatment, confirms that CTX significantly damage C2C12 derived myotubes. On the other hand pretreatment with metformin at 0.4, 1 and 5 mM, attenuates the severity of CTX damage (Figure 3E–3F).

### Metformin attenuates necrosis induced by cardiotoxin treatment

**Figure 4:**
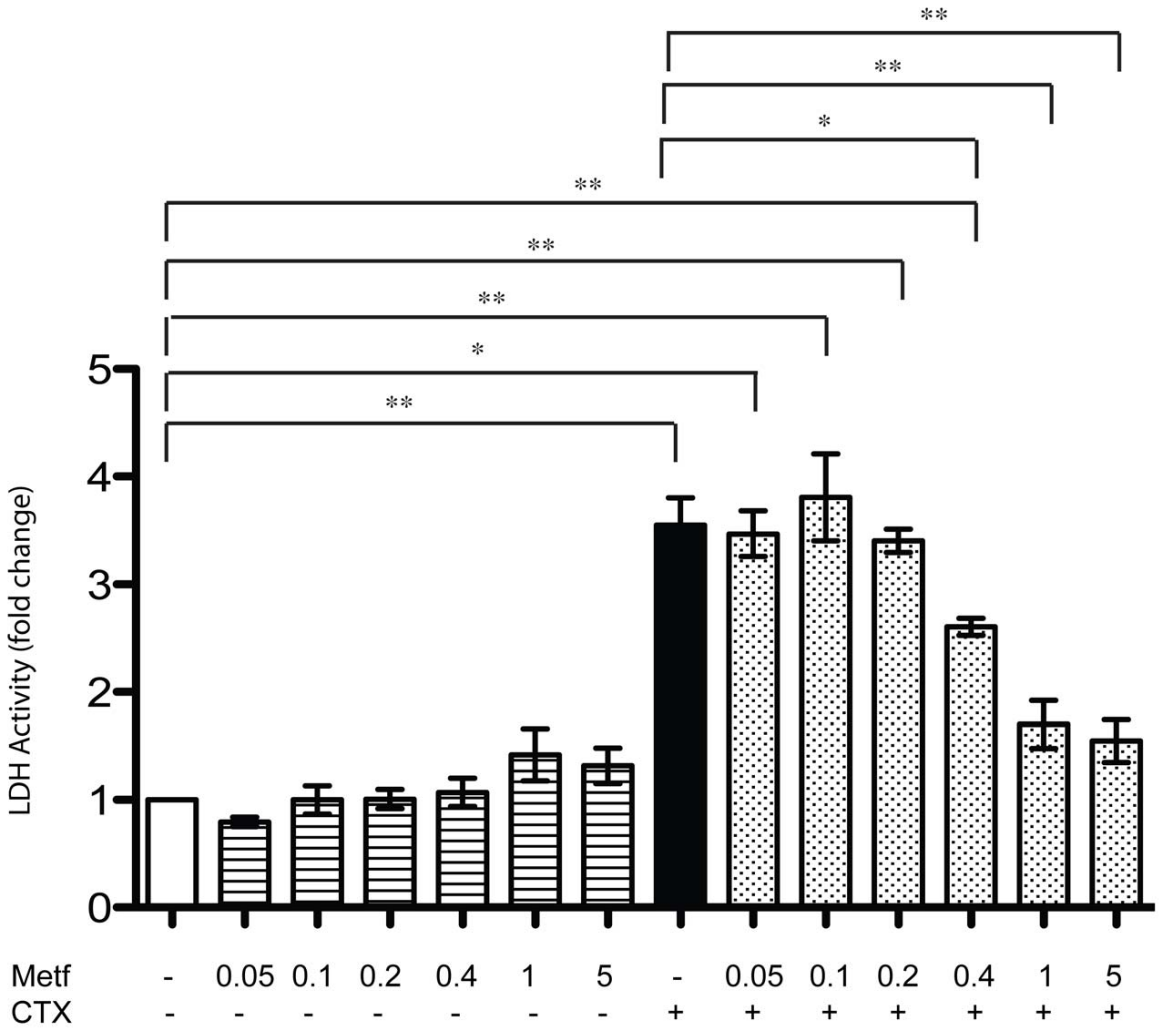
Metformin attenuates necrosis induced by cardiotoxin treatment. Differentiated C2C12 were pretreated with metformin at different concentrations (0.05, 0.1, 0.2, 0.4, 1, 5 mM) for 23 hours and then incubated with cardiotoxin (1 uM) for 1 h. Necrosis was estimated by measuring the LDH activity in the medium and by normalizing it with the total protein content. Data are expressed as fold change compared to controls and represent the mean of three experiments ± SE, *p<0.05, **p<0.01.

CTX injury induces necrosis [26]. Thus, we tested whether the protective effect observed after metformin treatment correlates with a decrease in the necrosis induced by CTX. Differentiated C2C12 cells were pre-treated with PBS or metformin at different concentrations for 23 hours and then injured with 1 µM CTX for 1 hour. Necrosis can be quantified in cell culture by measuring the release of the intracellular enzyme lactate dehydrogenase (LDH) [27]. Thus, we performed a Lactate-Dehydrogenase (LDH) assay after cardiotoxin treatment of C2C12 myotubes preincubated for 23 hours with different concentrations of metformin. While CTX significantly increases the release of LDH in the culture medium (Figure 4), metformin, on its own, had no significant effect in this assay. However, when C2C12 myotubes were pre-treated with high concentrations of metformin (0.4, 1, 5 mM) and then injured with CTX, the activity of LDH decreased significantly.

### Metformin counteracts the calcium influx induced by CTX

**Figure 5:**
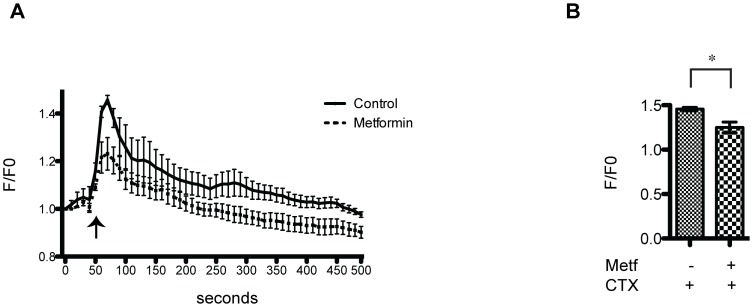
Metformin treatment reduces calcium influx induced by CTX. (A) Assessment of time dependent calcium influx induced by incubation with 1 µM CTX in differentiated C2C12 myotubes. Two samples of C2C12 differentiated myotubes were one treated with 5 mM metformin for 22 hours (dashed line) while the other was left untreated (solid lines). Cells were loaded with Fluo4-AM and emission of fluorescence light at 488 nm was monitored every 10 seconds under a fluorescence confocal microscope, with a 10× objective and 2× optical zoom for a total magnification of 20×, to monitor calcium uptake. 50 seconds after the acquisition start point CTX was added to the cell culture (arrow) and the changes in fluorescence monitored for a total of 500 seconds. Each condition was normalized to the measurements prior to stimulation. Data were expressed as fold change vs control. (B) The bar graph illustrates the mean of FLUO4-AM fluorescence intensity maximal peaks. Data represent the mean of three experiments ± SE, (*p<0.05).

CTX treatment induces elevated intracellular calcium levels, probably due to the influx from the extracellular milieu into C2C12 myotubes [18]. We investigated the effect of metformin on calcium flux in C2C12 differentiated myoblast. To this end C2C12 myotubes were loaded with the calcium sensitive dye Fluo4-AM and the analyses were performed either in time lapse by confocal microscope (Figure 5), or alternatively by FACS (Figure S3). In both cases we confirmed that CTX treatment significantly increases fluorescence, indicating elevated calcium influx. However the effect was appreciably attenuated when CTX damage was preceded by metformin (5 mM) treatment (Figure 5, Figure S3). These results suggest that metformin treatment counteracts the influx of calcium induced by CTX, thus limiting myotube damage and cell death.

## Discussion

Recent reports support the notion that perturbation of metabolism modulates muscle stem cell function and fate thereby influencing the recovery from injury after *in vivo* damage and the efficacy of engraftment of transplanted cells [4]. Short-term calorie restriction (CR) diet enhances stem cell activity and availability in skeletal muscle of young and old mice. The effect was also evident in skeletal muscle repair and in the contribution of metformin conditioned donor cells to regenerating muscle after transplantation. On the same theme, Jahnke and collaborators showed that AICAR, an inducer of the nutrient sensor AMPK, reduces muscle fatigability and improves performance of muscles from *mdx* mice by raising the level of PGC-1α, cytochrome c expression and by increasing mitochondrial biogenesis [5]. A metabolic reprogramming, that favors oxidative over glycolytic metabolism, is likely to play an important role in this processes. However, the molecular mechanisms underlying the link between metabolic alterations and the enhancement of myogenic activity of muscle stem cells remain unclear. Selsby and colleagues observed that enhancing PGC-1α expression by recombinant adeno-associated virus (AAV) infection, rescues dystrophic muscles and that a switch from fast- to slow-twitch muscle is involved [28].

Given that metformin is an ipoglycemic drug that impacts on cell metabolism by inducing AMPK and that metabolic reprogramming enhances muscle regeneration, we investigated the effect of metformin on skeletal muscle response to muscle injury. We induced muscle damage with an intramuscular injection of the snake venom fraction cardiotoxin (CTX), isolated from *Naja pallida*, This cytotoxic drug induces effects on the injured muscle (necrosis, inflammation, sarcolemma disruption etc) that are similar to those observed in muscular dystrophies (MDs) and inflammatory myopathies (IMs) [18].

We have observed that intraperitoneal injection of metformin enhanced the number of mitochondria rich, oxidative fibers in tibialis anterior (TA) (Figure 1A–1F) without substantially and consistently affecting the activity of the AMPK, ACC1 mTOR, RPS6 pathway when these readouts are measured after chronic exposure to metformin (Figure 1H). These results are consistent with the report of Suwa and colleagues, that metformin treatment enhances PGC-1α expression and mitochondrial biogenesis in the skeletal muscle, possibly via AMPK activation [23].

Besides, unexpectedly, we found that muscle sections from mice pre-treated with metformin and injured by CTX injection, showed a lower percentage of damaged areas when compared to the PBS injected control (Figure 2A–2E). Moreover, metformin untreated, CTX-injured mice at 5 days post injury, showed still the presence of degenerating fibers stuffed with inflammatory cells (Figure 2G), while metformin conditioned mice revealed an enhanced number of centronucleated regenerating myofibers (Figure 2L). Thus, our observation suggests that metformin treatment protects the skeletal muscle from CTX damage. To further characterize the mechanism of the observed protective effect, we extended our experiments to an *in vitro* model. We pre-treated C2C12 derived myotubes with metformin at different concentrations and then with CTX. The pretreatment with metformin did not affect either the number (Figure 3B) or the fusion index (Figure 3C) of C2C12 myotubes. Consistent with *in vivo* result we observed that the number of C2C12 myotubes injured with CTX after treatment with metformin were higher than in samples of myotubes that were not pretreated with metformin (Figure 3E–3F), thus confirming that metformin pre-treatment protects C2C12 myotubes from CTX damage. However the positive effect of metformin was evident only at concentrations of 0.4 mM or higher.

Since cardiotoxin (CTX) is known to induce necrosis both *in vitro* and *in vivo* [26], [29] we evaluated the protection against cytolysis offered by metformin, by measuring lactate dehydrogenase (LDH) activity released into the medium (Figure 4). The LDH assay showed that C2C12 myotubes treated with CTX undergo necrosis, while metformin conditioning significantly decreases LDH release from myotubes. This indicates that metformin can counteract the increased plasma membrane permeability induced by CTX.

CTX induced death involves calcium influx provoking an overload of calcium in the cytoplasm that triggers necrosis [18]. Calcium ion plays a fundamental role in skeletal muscle, as a main regulator and signaling molecule, acting as a messenger involved in processes ranging from activation of contraction to degradation of muscle cells. Thus skeletal muscle has developed a system that finely controls the concentration of calcium in the cytoplasm [30]. In the resting state the concentration of free calcium in the cytosol of a muscle cell is lower than the concentration in the extracellular fluid, creating a chemical gradient for calcium across the cellular membrane that determines a continuous passive diffusion of calcium into the muscle cells [31], [32]. In healthy muscle, when the calcium concentration in the cytosol increases, muscle cells store calcium in the sarcoplasmic reticulum [33] and in the mitochondria [34]. It is important for muscle cells to control calcium concentration in the cytosol because the overload of calcium could lead to the proteolysis of cellular constituents [35], by activating calpains [36]. In addition calcium concentration can affect membrane integrity, by activating the phospholipase A2 [37] that causes lipid peroxidation by increasing the production of ROS [38].

Since we observed that metformin reduces the membrane permeability caused by CTX, we wondered if the metformin protective effect could be attributed to an increased control of intracellular calcium homeostasis. To test this hypothesis, we measured the CTX induced increase in intracellular calcium and we tested if metformin pretreatment acted as countermeasure. In Figure 5, we show that CTX significantly induced calcium influx, while metformin counteracts the CTX effect. This effect on calcium flux is consistent with the observation that metformin treatment reduces calcium dependent-vitamin B_12_ absorption in patient with type 2 diabetes [39]. Metformin could affect the charge of the cell membrane since the biguanide molecule has a hydrophobic tail which allows the intercalation into the hydrocarbon core of the membrane, while the protonated group gives a positive charge to the cell surface which acts to repulse divalent cations such as calcium [40]. In support of the hypothesis that metformin could have a role in modulating calcium concentration in muscle it has been shown that metformin reduces migration and invasion of human fibrosarcoma cells by blocking Ca^2+^ influx and subsequently by suppressing matrix metalloproteinase-9 activation [41].

In conclusion, our work shows that metformin protects muscles from CTX induced damage both *in vivo* and *in vitro*. Metformin limits the CTX damage by protecting from necrosis, most likely by negatively modulating the CTX induced calcium influx.

Since muscle calcium overload occurs in a wide range of situations such as exercise [42], muscular dystrophy [43]–[45] and cachexia [46], countermeasures, such as membrane stabilizing agents or calpain inhibitors could help to prevent and reduce skeletal muscle damage. According to the obtained results, we can speculate that a long-term metformin treatment on dystrophy-affected patients could have a beneficial effect reducing/delaying dystrophic symptoms deferring muscle wasting.

## Supporting Information

Figure S1 **Metformin treatment enhances mitochondrial biogenesis.** (A,B) Immunofluorescence images of muscle fibers from metformin treated (200 mg/Kg) and untreated mice labeled with anti TOM20 antibody (green). Arrows point to TOM20 rich oxidative myofibers. (C,D) Higher magnification of samples prepared as in A and B. Nuclei are visualized (blue) by 4′,6-Diamidino-2-Phenylindole, Dihydrochloride (DAPI) counterstaining. (E) The bar graph represents the quantitation of the experiment in A, B, C, D. The average fraction of TOM20-rich fibers was estimated by inspection of five randomly selected fields of 3 different sections (magnification 20×) from each sample (n = 3). Statistical significance was evaluated by the Student's t-test (**p<0.01). Scale bar values: (A,B) 100 µm, (C,D) 25 µm. (F, G, H) The scatter plots show the densitometric analysis relative to figure 1H, each phosphorylation is normalized with the total content of the protein. (F) The graph shows the increment of ACC phosphorylation in Ser79, due to metformin treatment *in vivo*. (G) The graph shows the increment of AMPK phosphorylation in Thr172, due to metformin treatment *in vivo*. (H) The graph shows the decrement of RPS6 phopshorylation in Ser240/244, due to metformin treatment *in vivo*.
(TIF)

Figure S2 **Metformin treatment, in our experimental conditions, does not significantly affect the mTOR pathway in muscles.** Western blot analysis of total RPS6, 4EBP1 and their phosphorylation in total protein lysates from metformin-treated or control C2C12 derived myotubes. GAPDH is used as a loading control. Metformin does not alter the phosphorylation of mTOR downstreat proteins, RPS6 and 4EBP1.
(TIF)

Figure S3 **Analysis by flow cytometry of calcium flux.** The bar graph illustrates the mean of FLUO4-AM fluorescence intensity obtained by FACS analysis. The FLUO4-AM dye is directly proportional to the extracellular calcium influx. Cardiotoxin treatment significantly increases the fluorescent intensity of C2C12 myotubes stained with FLUO4-AM indicating an increment of calcium influx. Metformin treatment does not affect significantly calcium flux. The pretreatment of myotubes with metformin (5 mM) attenuates the increment of calcium influx upon CTX exposure. Data represent the mean of three experiments ± SD, (*p<0.05, **p<0.01, ***p<0.001).
(TIF)
