# Supplementary figures and images for: Metformin Protects Skeletal Muscle from Cardiotoxin Induced Degeneration
Source: PLoS One. 2014 Dec 2;9(12):e114018. doi: 10.1371/journal.pone.0114018 (PMC4252070; doi:10.1371/journal.pone.0114018)

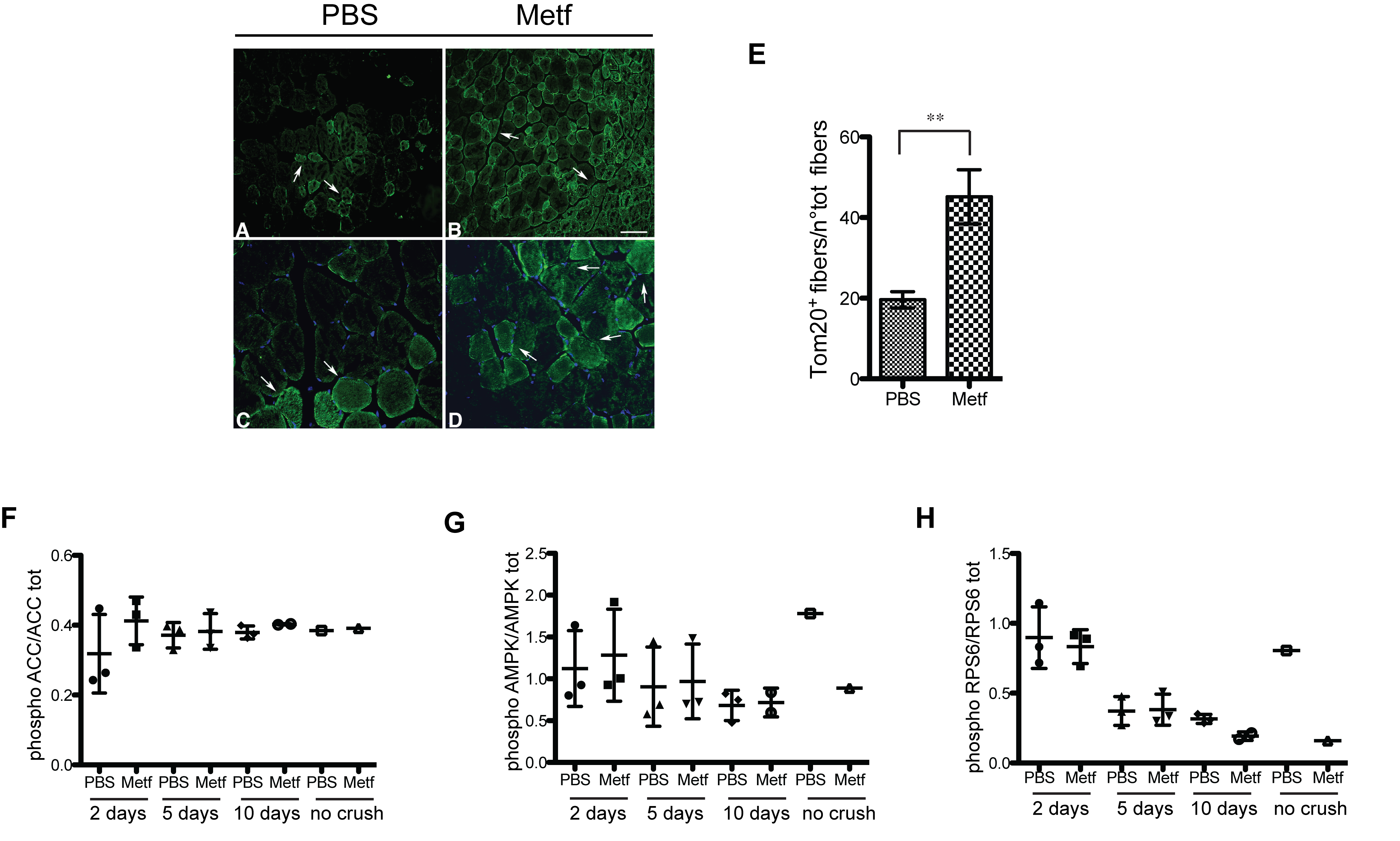

Supplement: Figure S1 — Metformin treatment enhances mitochondrial biogenesis. (A,B) Immunofluorescence images of muscle fibers from metformin treated (200 mg/Kg) and untreated mice labeled with anti TOM20 antibody (green). Arrows point to TOM20 rich oxidative myofibers. (C,D) Higher magnification of samples prepared as in A and B. Nuclei are visualized (blue) by 4′,6-Diamidino-2-Phenylindole, Dihydrochloride (DAPI) counterstaining. (E) The bar graph represents the quantitation of the experiment in A, B, C, D. The average fraction of TOM20-rich fibers was estimated by inspection of five randomly selected fields of 3 different sections (magnification 20×) from each sample (n = 3). Statistical significance was evaluated by the Student's t-test (**p<0.01). Scale bar values: (A,B) 100 µm, (C,D) 25 µm. (F, G, H) The scatter plots show the densitometric analysis relative to figure 1H, each phosphorylation is normalized with the total content of the protein. (F) The graph shows the increment of ACC phosphorylation in Ser79, due to metformin treatment in vivo. (G) The graph shows the increment of AMPK phosphorylation in Thr172, due to metformin treatment in vivo. (H) The graph shows the decrement of RPS6 phopshorylation in Ser240/244, due to metformin treatment in vivo. (TIF) [file pone.0114018.s001.tif]

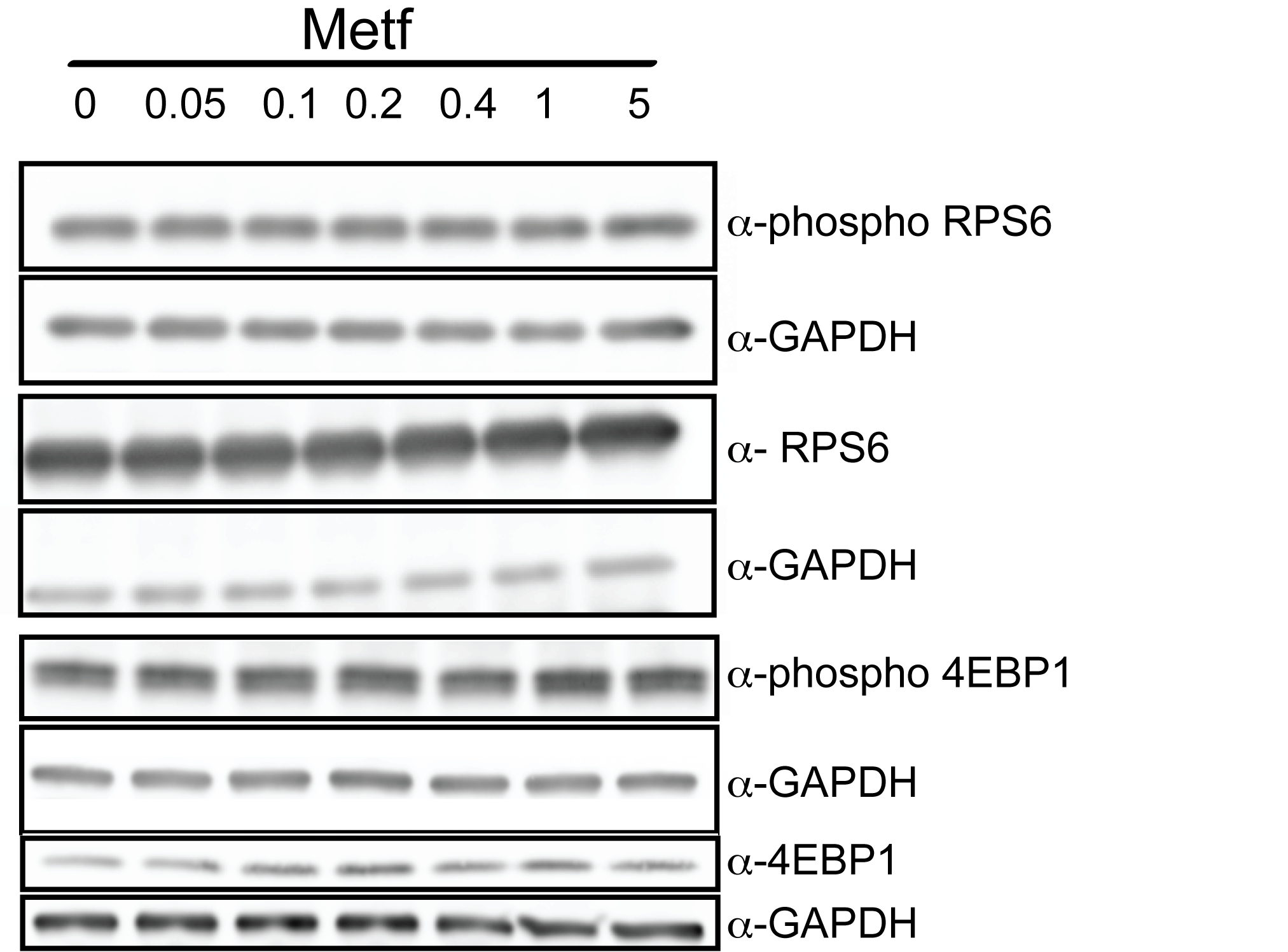

Supplement: Figure S2 — Metformin treatment, in our experimental conditions, does not significantly affect the mTOR pathway in muscles. Western blot analysis of total RPS6, 4EBP1 and their phosphorylation in total protein lysates from metformin-treated or control C2C12 derived myotubes. GAPDH is used as a loading control. Metformin does not alter the phosphorylation of mTOR downstreat proteins, RPS6 and 4EBP1. (TIF) [file pone.0114018.s002.tif]

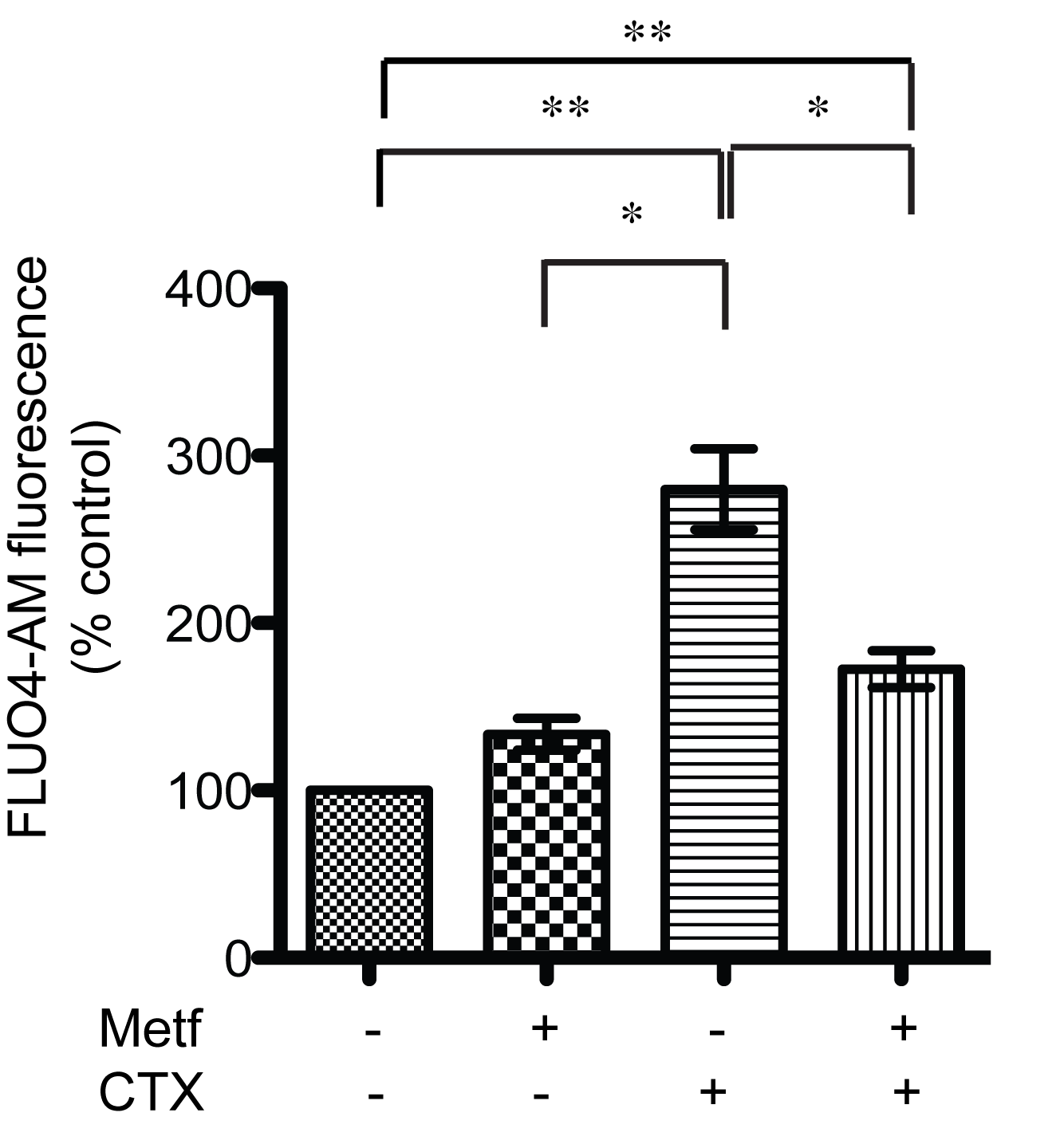

Supplement: Figure S3 — Analysis by flow cytometry of calcium flux. The bar graph illustrates the mean of FLUO4-AM fluorescence intensity obtained by FACS analysis. The FLUO4-AM dye is directly proportional to the extracellular calcium influx. Cardiotoxin treatment significantly increases the fluorescent intensity of C2C12 myotubes stained with FLUO4-AM indicating an increment of calcium influx. Metformin treatment does not affect significantly calcium flux. The pretreatment of myotubes with metformin (5 mM) attenuates the increment of calcium influx upon CTX exposure. Data represent the mean of three experiments ± SD, (*p<0.05, **p<0.01, ***p<0.001). (TIF) [file pone.0114018.s003.tif]
